# Which Facets of Mindfulness Protect Individuals from the Negative Experiences of Obsessive Intrusive Thoughts?

**DOI:** 10.1007/s12671-017-0854-3

**Published:** 2017-11-18

**Authors:** Lisa-Marie Emerson, Connor Heapy, Gemma Garcia-Soriano

**Affiliations:** 1 Clinical Psychology Unit, Department of Psychology, University of Sheffield, Floor F, Cathedral Court, 1 Vicar Lane, Sheffield, S1 2LT UK; 2 Facultad de Psicologia, University of Valencia, Valencia, Spain

**Keywords:** Obsessions, Intrusive thoughts, Mindfulness, Acceptance, OCD

## Abstract

Obsessive intrusive thoughts (OITs) are experienced by the majority of the general population, and in their more extreme forms are characteristic of obsessive–compulsive disorder (OCD). These cognitions are said to exist on a continuum that includes differences in their frequency and associated distress. The key factors that contribute to an increased frequency and distress are how the individual *appraises* and *responds* to the OIT. Facets of mindfulness, such as nonjudgment and nonreactivity, offer an alternative approach to OITs than the negative appraisals and commonly utilised control strategies that often contribute to distress. Clarifying the role of facets of mindfulness in relation to these cognitions offers a means to elucidate individual characteristics that may offer protection from distress associated with OITs. A sample of nonclinical individuals (*n* = 583) completed an online survey that assessed their experiences of OITs, including frequency, emotional reaction and appraisals, and trait mindfulness. The findings from a series of multiple regression analyses confirmed that specific facets of mindfulness relating to acting with awareness and acceptance (nonjudgment and nonreactivity) consistently predicted less frequent and distressing experiences of OITs. In contrast, the observe facet emerged as a consistent predictor of negative experiences of OITs. These findings suggest that acting with awareness and acceptance may confer protective characteristics in relation to OITs, but that the observe facet may reflect a hypervigilance to OITs. Mindfulness-based prevention and intervention for OCD should be tailored to take account of the potential differential effects of increasing specific facets of mindfulness.

Intrusive thoughts are a key characteristic of obsessive–compulsive disorder (OCD; American Psychiatric Association [APA] 2013) and have been defined as those spontaneous thoughts, images or impulses that are difficult to control, are disruptive and unwanted (Rachman 1981). Evidence also suggests that thoughts of a similar form are also experienced by a large proportion of the general population (Belloch et al. 2004; Langlois et al. 2000; Purdon and Clark 1993). The cognitive model of OCD is based on the understanding that obsessive intrusive thoughts (OITs) experienced by the general population exist at the opposite end of a continuum to those thoughts experienced by individuals with a diagnosis of OCD, differing in the frequency with which they are experienced and the distress they cause (Clark and Rhyno 2005). Individuals within clinical samples are likely to experience more extreme or severe forms of OITs, experience them more *frequently* and be more *distressed* by their occurrence, as they provoke negative emotional reactions (e.g. anxiety, sadness) and are experienced as difficult to control or neutralise (Berry and Laskey 2012). In a review of the continuum model, Berry and Laskey (2012) explain that the key factors that contribute to this increased frequency and distress are how the individual *appraises* and *responds* to the OIT. The evidence reviewed suggests that individuals with OCD and with subclinical scores on OCD appraise their OITs more dysfunctionally than nonclinical individuals, for example, by viewing them as important or feeling responsible for experiencing them. These OIT misinterpretations make them more emotionally disturbing increasing the efforts to control them. In addition, the review also highlighted differences in how individuals respond to their OITs: individuals with a diagnosis of OCD or with subclinical scores on OCD were more likely to engage in a variety of cognitive (e.g. thought suppression, covert restructuring, worry, mental compulsions) and behavioural (e.g. washing, ordering) strategies, to dismiss the frequency of the OIT or to overcome the anxiety that was provoked.

As a cognitive control strategy, thought suppression, or actively trying to remove the thought (Wegner 1989), is laden with problems. A large number of studies have demonstrated that suppression is ineffective at removing intrusive thoughts, in both clinical and nonclinical samples (e.g. Grisham and Williams 2009; Marcks and Woods 2007; Purdon et al. 2007). For example, Purdon et al. (2007) tracked the OITs experienced in a sample of individuals diagnosed with OCD as well as the corresponding response strategies they used, and found that only 11% of suppression attempts were successful in removing a target thought. Furthermore, greater frequency and duration of suppression episodes correlate with greater OCD symptom severity in both clinical (Purdon et al. 2007) and nonclinical samples (Clark and Purdon 2009).

Mindfulness may offer an alternative response to OITs; in particular, the nonjudgmental acceptance of thoughts (Baer 2003) runs counter to the judgments thought to be central to the maintenance of OCD. Mindfulness has been described as a temporary and evoked state of being, as well as a more stable individual trait or characteristic (Brown and Ryan 2003). The most commonly used definitions and operationalisations of mindfulness refer to receptive awareness and attentional focus (Bishop et al. 2004; Brown and Ryan 2003; Baer et al. 2006). The attentional component of mindfulness involves a self-regulated directing of attention to the present moment experience and is supported by an attitudinal component of openness and curiosity toward those experiences that arise. Baer et al. (2006) determined five specific facets of mindfulness. The attentional component was captured by facets relating to *acting with awareness*, and *observing* present moment experiences, whilst the attitudinal component of mindfulness was captured by facets relating to the suspension of judgment (*nonjudgment*) and automatic reactions (*nonreactivity*) in relation to experiences. The fifth factor relates to the tendency to *describe* one’s experience, for example, to put experiences into words.

The five facets of mindfulness (Baer et al. 2006) have been shown to be differentially related to psychopathology presentations. Cash and Whittingham (2010) reported that, in a mixed sample of meditators and undergraduate students, higher levels of nonjudgment and acting with awareness were predictive of lower levels of psychopathology, with nonjudgment being specifically related to symptoms of anxiety. In relation to OCD, previous research has supported the importance of the nonjudgment facet of mindfulness in differentiating between people with OCD and healthy controls. For example, Didonna (2009) reported that individuals with OCD scored lower on the nonjudgment facet, as well as acting with awareness and nonreactivity, compared to healthy controls. Crowe and McKay (2016) similarly reported that nonclinical participants who scored over the clinical cut-off for OCD also scored lower on nonjudgment, acting with awareness, and describe facets. Crowe and McKay interpret their findings in relation to the opposition of these facets to the presentation of OCD. In particular, the findings around the nonjudgment and acting with awareness are set in the context of an individual’s response to OITs. A pre-occupation with OITs runs counter to the acting with awareness facet of mindfulness; similarly, dysfunctional appraisals of OITs are inherently judgments of those experiences and oppose the nonjudgment facet of mindfulness. In this sense, trait mindfulness represents an individual’s tendency to respond to thoughts and feelings with acceptance, which is counter to the negative appraisals which commonly contribute to distress in relation to OITs. Therefore, individual differences in trait mindfulness may be of particular relevance to understanding how more positive or neutral appraisals of thoughts and feelings relate to the experience OITs.

Given the prevalence of OITs across the general population, exploring the role of mindfulness facets in relation to these cognitions offers a means to elucidate individual characteristics that may be related to OITs. As such, this would also expand our current understanding of the proposed continuum from intrusive thoughts to obsessions, in a direction related to a positive or neutral stance of toward OITs. The cognitive model of OCD, and previous research, would suggest that the attentional facet of acting with awareness may be related to reduced distress. The ability to focus one’s attention on the task at hand may make it less likely that OITs will intrude into consciousness, or that they would be less prominent in mind. In addition, the attitudinal component (nonjudgment and nonreactivity) appears to also be a key factor in distinguishing individuals with OCD; avoiding criticism of experiences may enable the individual to disengage from automatic reactions, which in the context of OITs may include dysfunctional appraisals (e.g. responsibility; Salkovskis 1985) or maladaptive coping strategies (e.g. thought suppression). The combined effect of these key facets of awareness and acceptance may allow intruding OITs to be dismissed and attention re-focused on the primary object or task.

In contrast, the observe facet may be related to a vulnerability in relation to OCD. Although this facet is understood in the context of the awareness component of mindfulness, it is distinguished from the facet of acting with awareness by the focus on noticing internal and external stimuli. In the context of OITs, higher levels of the observe facet may indicate an increased awareness of OITs, and increased vigilance to external triggers. Indeed, Crowe and McKay (2016) reported no deficits in the observe facet in the subclinical OCD group compared to healthy controls; they suggest that individuals with OCD may actually be ‘hyper-aware’ of specific internal experiences, such as OITs.

The main aim of the present investigation was to determine the relationship between OITs and the five facets of mindfulness in a nonclinical sample. We predicted that the most important facets would be those relating to awareness and an attitude of acceptance. Specifically, we predicted that acting with awareness, nonjudgment and nonreactivity would emerge as the most common and important predictors of OIT experience (specifically, lower frequency of OITs, less severe emotional reactions and fewer dysfunctional appraisals and strategies). We hypothesised that all three facets would predict lower frequency of OITs, whilst the attitudinal components (nonjudgment and nonreactivity) would predict the subsequent response to OITs (less severe emotional reactions and fewer dysfunctional appraisals and strategies). In contrast, we hypothesised that the ‘observe’ facet would predict a higher frequency of OITs and an increase in dysfunctional appraisals and emotions, and maladaptive strategies.

## Method

### Participants

Five hundred and eighty-three participants were recruited over two time periods from the University of Sheffield’s volunteer list via e-mail invitation. All participants were therefore affiliated with the University of Sheffield, e.g. staff, students, alumni. Average age of the participants was 25.85 years (*SD* = 9.67) and 69.5% were female. Ethical approval was granted by the university’s psychology ethics committee.

### Procedure

All participants completed an anonymous, online survey of questionnaires. The survey was administered using the survey platform Qualtrics. Participants completed the questionnaires in the order presented below. Participants who completed all questionnaires were entered into a cash prize draw. All participants provided informed consent prior to participation.

### Measures

A *demographic questionnaire* assessed gender and age.

The Obsessive–Compulsive Inventory–Revised (OCI-R; Foa et al. 2002) is an 18-item self-report measure used to assess OCD symptoms. Participants were presented with a list of symptoms (e.g. ‘I feel compelled to count while I am doing things’) and were required to indicate on a scale from 0 (‘not at all’) to 4 (‘extremely’) how distressed each symptom has made them in the past month. Symptoms were assessed over six domains: (1) washing, (2) checking, (3) ordering, (4) obsessing, (5) hoarding and (6) mental neutralising. Total OCI-R score is calculated by summing all items. The optimal score for distinguishing between nonclinical individuals and individuals with OCD is 21, such that scores of ≥ 21 are considered clinically meaningful (Foa et al. 2002). The OCI-R has demonstrated good internal consistency (*α* = .89) and test-retest reliability (*r* = .84) in nonclinical participants (Foa et al. 2002). Additionally, the OCI-R has demonstrated good convergent and divergent validity in a nonclinical, university sample, correlating higher with measures of OCD than measures of depression and worry (Hajcak et al. 2004). Cronbach’s alpha for the current sample was excellent (*α* = .89).

The Obsessional Intrusive Thoughts Inventory (original Spanish version: ‘Inventario de Pensamientos Intrusos Obsesivos’, INPIOS; García-Soriano 2008; García-Soriano et al. 2011; García-Soriano and Belloch 2013) is a two-part measure of OITs frequency, emotional reactions, appraisals, difficulty controlling and control strategies. For part 1 of the INPIOS, participants were presented with a list of 48 OITs (e.g. ‘Without being provoked I have had a mental intrusion of saying something inappropriate, bothering or insulting a stranger’) and were required to indicate on a scale from 0 (‘never’) to 6 (‘always’) how frequently they had experienced each OIT across six domains: (1) aggression; (2) sexuality, religion and immorality; (3) contamination; (4) doubts, mistakes and necessity to check; (5) symmetry and order and (6) superstition. Total scale and subscale scores for part 1 of the INPIOS are calculated by dividing the total score/subscale score by the number of items with a frequency ≥ 1 (see García-Soriano et al. 2011). Part 1 of the INPIOS has demonstrated high internal reliability (*α* = .94) and test-retest reliability (*ICC* = .97) in a nonclinical sample (García-Soriano et al. 2011). In addition, the INPIOS part 1 has demonstrated good convergent and divergent validity in individuals diagnosed with OCD, with total score correlating more highly with measures of OCD than depression, anxiety and worry. Cronbach’s alpha for the current sample was excellent (*α* = .95).

For part 2 of the INPIOS, participants were asked to indicate which of the more frequent OITs from part 1 they experienced as the most unpleasant in the last 3 months. This most unpleasant OIT is selected to be analogous to clinical obsessions, which are by definition frequent and distressing (e.g. American Psychiatric Association 2013). Participants then answered subsequent questions in relation to this selected OIT, assessing emotional reactions to (e.g. ‘How *unpleasant* is the intrusive thought?’), difficulty controlling (e.g. ‘How successful are you at controlling or suppressing the intrusive thought?’) and dysfunctional appraisals (e.g. ‘How important is the intrusive thought for you?’) from the past month. Participants were required to answer the questions on a scale from 0 (‘not at all’) to 4 (‘extremely’). Finally, participants were presented with a range of control strategies and were required to indicate how often they used each strategy (e.g. ‘I try to relax’) on a scale from 0 (‘never’) to 4 (‘always’). These strategies are grouped in four empirically derived factors and one independent item (García-Soriano 2008a): (1) general strategies to control anxiety (i.e. cognitive restructuring, reappraisal, reassurance from others, self-reassurance and relaxation); (2) cognitive thought control strategies (i.e. mental compulsion, thought stopping, self-punishment, avoidance, thought suppression efforts, worry, attempts to control and concealment); (3) distraction (cognitive and behavioural); (4) compulsions (i.e. washing, checking, ordering and repeating). Subscale scores for part 2 of the INPIOS were calculated by summing each subscale item. A total score including the different scales was calculated to appraise the frequency of control strategies used. Part 2 of the INPIOS has demonstrated good internal consistency (*α* = .76–.91) and good retest reliability (*r* = .78–.89; García-Soriano and Belloch 2013). Cronbach’s alphas for the current sample were acceptable for all subscales (*α* = .75–.85).

The Brief Mindfulness Measure (BMM; Berry et al. 2010) is a 10-item self-report measure used to assess trait mindfulness. The BMM was developed as a shorter version of the Five Facet Mindfulness Questionnaire (FFMQ; Baer et al. 2006). The BMM has shown a similar structure to the FFMQ, with five factors: (1) noticing inner experience (observe), (2) ability to label inner experiences (describe), (3) acting with awareness, (4) nonjudgment of inner experience (nonjudgment) and (5) nonreactivity to inner experience (nonreactivity). Participants were presented with a list of statements (e.g. ‘even when I’m feeling terribly upset, I can find a way to put it into words’) and were required to indicate on a scale from 1 (‘never or rarely true’) to 5 (‘very often or always true’) how often that statement applied to them. Total score of the BMM is calculated by summing all items. Subscale scores of the BMM are calculated by summing all subscale items. The BMM has demonstrated high test-retest reliability (*r* = .86), adequate split-half reliability (*r* = .63) but low internal reliability (*α* = .54; Berry et al. 2010). However, this low internal reliability is unsurprising given the five-factor structure of the BMM and suggests that it may be more appropriate to interpret subscales rather than scale total. The BMM also demonstrated good convergent validity, with BMM total score correlating highly with the total score of the combined KIMS and FFMQ items (*r* = .87). Cronbach’s alpha for the current sample was acceptable (*α* = .67).

### Data Analyses

All data analyses were carried out using IBM SPSS version 24 for Mac. Preliminary analyses assessed differences between participants who scored under the clinical cut-off on the OCI-R (< 21; ‘nonclinical’) and those that scored over the clinical cut-off (≥ 21; ‘subclinical’) on age, gender, intrusive thought frequency (INPIOS part 1) and trait mindfulness (BMM) by a series of between-group *t* tests and analysis of co-variance (ANCOVA) test as appropriate.

The main analyses investigated the relationship between OIT experiences (frequency, emotional reaction, difficulty controlling, dysfunctional appraisals and control strategies) and facets of mindfulness. Initial Pearson correlation coefficients were calculated for INPIOS and BMM totals and subscales. Threshold conventions were used to interpret the strength of associations (i.e. small = .10, medium = .30, large = .50; Rosenthal and Rosnow 2007). Medium and significant correlations between BMM total and INPIOS subscales were investigated further in a series of multiple regression analyses to determine which facets of mindfulness were most predictive of intrusive thought experiences. Facets of mindfulness were entered as independent variables in a stepwise fashion to predict intrusive thought frequency (INPIOS part 1), and frequency of participants’ most unpleasant intrusive thought. In a series of multiple regression analyses on INPIOS part 2, emotional reactions, difficulty controlling, dysfunctional appraisals and control strategies were entered as dependent variables. BMM subscales were entered simultaneously using stepwise method in step 2, after controlling for the frequency of the most unpleasant OIT (introduce method) in step 1. A Bonferroni correction was applied to the *p* values to account for the number of regression analyses performed on the same dataset, and protect against Type 1 error. As such, a more stringent *p* value of .007 (.05 divided by 7) was applied to the interpretation of the significance of regression models.

Data were inspected to ensure no violation of the assumptions of regression analyses. Pearson product-moment correlation coefficients between the independent variables were inspected for evidence of multi-collinearity. Durbin–Watson statistic was used to consider if auto-correlations were present in the variables. Visual inspections of the distribution of residuals, using QQ and PP plots, were carried out for each regression model to assess normality and homoscedasticity. Any indication of non-normality was followed up by inspection of the skewness and kurtosis for respective variables.

## Results

Overall, the sample of participants scored a mean of 17.38 (*SD* = 11.7) on the OCI-R. The mean total number of OITs endorsed on the INPIOS across the sample was 26.96 (10.78), with an average frequency (total frequency across thoughts divided by total number of thoughts endorsed) mean of 2.21 (*SD* = .73). Participants scored a mean of 26.87 (*SD* = 5.28) on the BMM.

Table 1 presents the bivariate correlations between BMM and INPIOS totals and subscales. Medium correlations were observed between total BMM and INPIOS frequency and emotional reaction, difficulty controlling and dysfunctional appraisals subscales. A medium correlation was observed between BMM total and the frequency of control strategies; however, a medium correlation held only between BMM total and cognitive thought control strategies when INPIOS subscales were investigated.

**Table 1:** Pearson product-moment correlation coefficients for pairwise correlations between BMM and INPIOS totals and subscales

	BMM					
INPIOS	Total	Observe	Describe	Act with awareness	Nonjudgment	Nonreactivity
Average frequency of OITs (*n* = 583)	− .401**	.189**	− .169**	− .352**	− .478**	− .156**
Frequency of most unpleasant OIT (*n* = 415)	− .343**	.041	− .147**	− .258**	− .332**	− .169**
Emotional reaction	− .406**	.167**	− .199**	− .220**	− .430**	− .283**
Difficulty controlling	− .422**	.057	− .149*	− .330**	− .312**	− .330**
Dysfunctional appraisals	− .427**	.151**	− .152**	− .266**	− .479**	− .280**
Frequency of control strategies	− .365**	.141*	− .112*	− .259**	− .443**	− .199**
Anxiety general control strategies	− .135**	.108*	.000	− .096*	− .224**	− .082
Cognitive thought control strategies	− .370**	.139*	− .225**	− .232**	− .440**	− .132**
Distraction strategies	− .259**	.029	− .112*	− .175**	− .234**	− .162**
Compulsions	− .155**	.078	− .069	− .095*	− .166**	− .110*

*INPIOS* Obsessional Intrusive Thoughts Inventory, *BMM* Brief Mindfulness Measure

*p < .05; **p < .001

An independent samples *t* test demonstrated that the nonclinical group were significantly older (*M =* 26.61, *SD =* 10.29*)* than the subclinical group (*M =* 24.26, *SD =* 8.04*), t*(581) *= −* 2.77, *p* = .006. Additionally, a chi-square analysis demonstrated no significant gender differences across groups (*χ* ^2^(2) = 1.24, *p* = .54).

In a series of ANCOVA tests controlling for group differences in age, significant differences (all *p* < .001) were observed between the nonclinical (*n* = 394; OCI-R ˂ 21) and subclinical (*n* = 189; OCI-*R* ≥ 21) groups on INPIOS part 1 and BMM total score and facets (see Table 2). The subclinical group reported a greater average frequency of OITs than the nonclinical group. In addition, the subclinical group reported (i) more negative emotional reaction to OITs, (ii) more difficulty controlling OITs (iii) more dysfunctional appraisals of OITs and (iv) carrying out more control strategies (anxiety control strategies, cognitive thought control strategies, distraction) than the nonclinical group.

**Table 2:** Differences between nonclinical and subclinical groups (based on OCI-R scores) on obsessive intrusive thoughts (INPIOS) and mindfulness (BMM)

	OCI-R ˂ 21 *n* = 394 Mean (SD)	OCI-*R* ≥ 21 *n* = 189 Mean (SD)	*F* statistic (581)*	Cohen’s *d*
INPIOS part 1 (average frequency)	1.93 (.56)	2.77 (.73)	220.14	− 1.280
INPIOS part 2				
Emotional reaction	7.8 (4.6)	11.86 (4.91)	89.71	− .844
Difficulty controlling	5.04 (2.71)	7.41 (2.44)	102.33	− .919
Dysfunctional appraisal	11.99 (6.57)	18.78 (6.62)	129.91	− 1.030
Cognitive control strategies	6.08 (4.73)	10.43 (6.69)	73.83	− .749
Anxiety general control strategies	5.24 (2.90)	7.79 (3.39)	87.76	− .808
Distraction strategies	4.11 (1.92)	5.11 (1.89)	31.05	− .524
BMM total score	24.01 (4.73)	28.23 (4.97)	86.58	− .861
Describe	5.89 (2.04)	6.61 (1.82)	13.04	− .375
Nonjudgment	4.49 (1.88)	6.40 (2.93)	118.18	− .995
Nonreactivity	5.45 (2.08)	6.37 (2.03)	24.84	− .452
Act with awareness	4.69 (1.85)	5.77 (1.84)	40.48	− .588
Observe	3.48 (1.12)	3.07 (1.16)	16.17	.354

*OCI-R* Obsessive Compulsive Inventory–Revised, *INPIOS* Obsessional Intrusive Thoughts Inventory, *BMM* Brief Mindfulness Measure, *OCI-R* Obsessive Compulsive Inventory–Revised

*All results significant to *p* < .001

In comparison with the nonclinical group, the subclinical group reported significantly lower scores on all the BMM facets, except for the observe subscale, which were higher, with medium-high effect sizes (Cohen 1988). See Table 2.

### Data Assumptions

The maximum Pearson’s correlation coefficient between independent variables was *r* = .36; none of the predictors were multi-collinear. Durbin–Watson statistics close to 2 indicated no auto-correlation of variables (range from 1.82 to 1.98). A visual inspection of QQ and PP plots indicated normality for all variables. The exception was for INPIOS cognitive thought control strategies; however, statistical values for skewness (1.01) and kurtosis (1.06) indicated that data were within the acceptable limits of ± 2 (Field 2009).

### Which Facets of Mindfulness Predict Experiences of OITs?

#### Frequency of All OITs

A stepwise multiple regression determined which mindfulness facets predicted the frequency of OITs (IVs: BMM subscales; DV: INPIOS total frequency). The final model (see Table 3) confirmed that nonjudgment, act with awareness and observe significantly predicted the total frequency of OITs (*F*(3, 578) = 75.55, *p* < .001) and explained 28% of the variance in frequency of OITs with nonjudgment accounting for the greatest proportion of variance in the model (23%). Act with awareness accounted for an addition 4% of variance, and observe accounted for 2% of unique variance. Nonjudgment and act with awareness showed negative relationships with frequency of OITs, whereas observe showed a positive relationship.

**Table 3:** Stepwise regression model, predicting frequency of obsessional intrusive thoughts (INPIOS part 1) from mindfulness facets (BMM subscales)

BMM facets	Unstandardised coefficients		Standardised coefficients
	*B*	*SE b*	*β*
Step 1			
Constant	3.16	.078	
Nonjudgment	− .17	.013	− .48*
Step 2			
Constant	3.44	.092	
Nonjudgment	− .14	.013	− .40*
Act with awareness	− .08	.015	− .21*
Step 3			
Constant	3.15	.12	
Nonjudgment	− .14	.01	− .39*
Act with awareness	− .08	.01	− .20*
Observe (1 item)	.08	.02	.13*

Unstandardised coefficients: *B*, *SE b.* Standardised coefficients*: β*

*BMM* Brief Mindfulness Measure, *INPIOS* Obsessional Intrusive Thoughts Inventory

*p < .001

#### Frequency of the Most Unpleasant OIT

Four hundred and fifty-one participants endorsed an OIT as the most unpleasant intrusive thought during the last 3 months and completed part 2 of the INPIOS (the remaining participants did not endorse experiencing an unpleasant thought). The most unpleasant OIT is selected to be the analogue to a clinical obsession, as it is not only one of the most frequent OITs from part 1, but also the one that provokes higher unpleasantness. A stepwise multiple regression determined which mindfulness facets predicted the frequency of the most unpleasant OIT. The final model (see Table 4) confirmed that nonjudgment, act with awareness and nonreactivity significantly predicted frequency of the most unpleasant OIT (*F*(1, 450) = 55.49, *p* < .001) and explained 15% of total variance. The nonjudgment subscale accounted for the greatest proportion of variance in the model (11%); act with awareness accounted for an additional 3% of variance, and nonreactivity an additional 1%. All significant predictors showed negative relationships with the frequency of the most unpleasant OIT.

**Table 4:** Stepwise regression model, predicting the frequency of most unpleasant OIT (INPIOS) from mindfulness facets (BMM)

BMM facets	Unstandardised coefficients		Standardised coefficients
	*B*	*SE b*	*β*
Step 1			
Constant	4.474	.190	
Nonjudgment	− .234	.031	− .332
Step 2			
Constant	4.974	.231	
Nonjudgment	− .196	.033	− .278
Act with awareness	− .134	.036	− .171
Step 3			
Constant	5.310	.277	
Nonjudgment	− .183	.033	− .258
Act with awareness	− .131	.036	− .167
Nonreactivity	− .071	.032	− .098

Unstandardised coefficients: *B*, *SE b.* Standardised coefficients*: β*

*BMM* Brief Mindfulness Measure, *INPIOS* Obsessional Intrusive Thoughts Inventory

#### Emotional Reactions, Difficulty Controlling and Dysfunctional Appraisals

A series of hierarchical regression analyses were computed to determine which mindfulness facets contribute to predicting the emotional reactions, difficulty controlling and dysfunctional appraisals in relation to the unpleasant OIT. Frequency of the most unpleasant OIT was entered in step 1 (enter method), following which mindfulness subscales were entered in step 2 (stepwise method). The final models for each analysis are presented in Table 5.

**Table 5:** Final models from stepwise regression analyses predicting emotional reactions, difficulty controlling, dysfunctional appraisals and frequency of control strategies (INPIOS part 2) from mindfulness facets (BMM subscales)

Predictors	Emotional reactions	Difficulty controlling	Dysfunctional appraisals	Frequency control strategies
Frequency of OIT				
*B*	.337	.596	1.062	1.078
*SE b*	.145	.078	.199	.361
*β*	.101	.322	.222	.133
Nonjudgment				
*B*	− .797	− .124	− 1.205	− 1.929
*SE b*	.104	.057	.143	.262
*β*	− .339	− .095	− .357	− .337
Nonreactivity				
*B*	− .483	− .316	− .595	− .580
*SE b*	.102	.054	.140	.250
*β*	− .201	− .235	− .172	− .09
Act with awareness				
*B*	–	− .279	–	− .627
*SE b*	–	.061	–	.280
*β*	–	− .193	–	− .099
Observe				
*B*	.586	–	.690	.973
*SE b*	.181	–	.247	.444
*β*	.134	–	.110	.091

Unstandardised coefficients: *B*, *SE b.* Standardised coefficients*: β*

*BMM* Brief Mindfulness Measure, *INPIOS* Obsessional Intrusive Thoughts Inventory

Regarding the emotional reaction evoked by the most unpleasant OIT, the final model accounted for 25% of the variance [*F*(4, 446) = 37.34, *p* < .001]. Frequency of the most unpleasant OIT accounted for 6% of the variance and was significantly and positively related to emotional reaction. The individual BMM predictors that entered the final model were nonjudgment, nonreactivity and observe facets. Nonjudgment accounted for the greatest proportion of variance (14%), nonreactivity accounted for an additional 3% of the variance and observe accounted for 2% of unique variance. Nonjudgment and nonreactivity showed significant negative associations with emotional reaction, whereas, the observe facet showed a significant positive association.

Regarding the difficulty controlling the most unpleasant OIT, the final model accounted for 31% of the variance [*F*(4, 446) = 51.02, *p* < .001]. Frequency of the most unpleasant OIT accounted for 20% of variance in the final model, with a positive and significant association with difficulty controlling the unpleasant OIT. The individual BMM predictors that entered the final model were nonreactivity, act with awareness and nonjudgment. Of the BMM predictors, nonreactivity accounted for the greatest proportion of variance (7%); act with awareness accounted for 4% of unique variance and nonjudgment 1% of unique variance. Each of the BMM facets showed significant negative associations with difficulty controlling the most unpleasant OIT.

Finally, regarding the dysfunctional appraisals associated with the most unpleasant OIT, the final model accounted for 32% of the variance [*F*(4, 446) = 52.30, *p* < .001]. Frequency of the most unpleasant OIT accounted for 14% of the variance in the final model, with a positive and significant association with dysfunctional appraisals. The individual BMM predictors that entered the final model were nonjudgment, nonreactivity and observe. Of the BMM predictors, nonjudgment accounted for the greatest proportion of unique variance (14%), nonreactivity accounted for 3% of unique variance and the observe facet accounted for 1% of variance. Nonreactivity and nonjudgment showed significant positive associations with dysfunctional appraisals, whereas the observe facet showed a significant positive association.

#### Use of Control Strategies

Moderate and significant correlations between the BMM and frequency of all control strategies (total for INPIOS part 2b) were investigated further by hierarchical regression analysis. After controlling for the frequency of the OITs (step 1—introduce method), mindfulness subscales were entered in step 2 (stepwise method). The final model (Table 5) accounted for 24% of the variance [*F*(5, 445) = 29.06, *p* < .001]. The frequency of the most unpleasant OIT accounted for 9% of the variance in frequency of control strategies. The individual BMM predictors that entered the final model were nonjudgment, act with awareness, nonreactivity and observe. Nonjudgment accounted for the greatest proportion of unique variance (14%); act with awareness accounted for 1% of variance; nonreactivity and observe each accounted for 08% unique variance. Nonjudgment, act with awareness and nonreactivity showed significant negative associations with control strategies, whereas the observe facet showed a significant positive association.

Furthermore, the moderate and significant relationship observed between the BMM and cognitive thought control strategies was investigated further. A linear regression analysis was conducted with thought control strategies as the dependent variable. After controlling for the frequency of the OITs (step 1—introduce method), mindfulness subscales were entered in step 2 (stepwise method). The final model accounted for 30% of the variance [*F*(4, 446) = 49.09, *p* < .001]. The frequency of the most unpleasant OIT accounted for 19% of variance in thought control strategies. The individual BMM predictors that entered the final model were the nonjudgment (Δ*R* ^2^ = .10), describe (Δ*R* ^2^ = .009) and observe. Of the BMM predictors, nonjudgment accounted for the greatest proportion of variance (10%); describe accounted for .09% unique variance; observe accounted for .08% unique variance. Nonjudgment and describe showed significant negative associations with thought control strategies, whereas the observe facet showed a positive and significant association.

## Discussion

The present study aimed to investigate the predictive relationship between facets of trait mindfulness (e.g. observing, nonjudgment) and aspects of OIT experience (e.g. frequency, control strategies, appraisals). Consistent with previous research, nonclinical individuals scored higher on overall trait mindfulness than individuals with subclinical OCD. As hypothesised, three facets of mindfulness (nonjudgment, nonreactivity and act with awareness) predicted fewer OITs (total/most upsetting) and less difficulty controlling OITs. Two of the same mindfulness facets (nonjudgment and nonreactivity) predicted less severe emotional reactions to OITs and less dysfunctional OIT appraisals. Consistent with our predictions, the attentional facet of act with awareness was more important in predicting the occurrence of OITs (frequency), than subsequent response (emotional reactions or dysfunctional OIT appraisals). In addition, as hypothesised, three mindfulness facets (nonjudgment, nonreactivity and act with awareness) predicted less use of control strategies, with the strongest correlation observed between mindfulness and cognitive control strategies. In line with our predictions, the observe facet of mindfulness (a tendency to notice internal and external stimuli) emerged as a consistent predictor of negative experiences of OITs.

The finding that nonclinical individuals score higher on overall trait mindfulness than individuals with subclinical OCD is in line with previous findings (e.g. Crowe and McKay 2016). This is not surprising when considering that many of the beliefs (e.g. thought-action fusion) and responses (e.g. suppression) that are central to OCD are antithetical to a state of mindfulness (e.g. thoughts are not actions, accept thoughts without reaction, Crowe and McKay 2016). In fact, nonclinical individuals scored higher on each individual facet of mindfulness than those with subclinical OCD, except the observe facet. The largest difference between the two groups was on the nonjudgment facet, which has also been found previously (Crowe and McKay 2016). Again, this finding is not surprising when considering that a key distinguishing feature between individuals with OCD and those without is the way that they judge their OITs (Rachman 1997, 1998; Salkovskis 1985).

The difference between the nonclinical individuals and those with subclinical OCD on the observe and nonreactivity facets conflicts with findings from a previous study (Crowe and McKay 2016). In the present study, the difference in scores on the observe facet was the smallest of all comparisons, so it is not particularly surprising that a difference between nonclinical individuals and those with subclinical OCD was not found previously. In the case of the nonreactivity facet, the difference may be due to the mindfulness measures chosen by each study (FFMQ; Baer et al. 2006 vs BMM; Berry et al. 2010). The FFMQ assesses nonreactivity with questions such as ‘when I have a distressing thought, I feel calm soon after’. Individuals with subclinical OCD may have indicated that this is true for them because they carried out a compulsion after the OIT, which temporarily reduced anxiety levels (Crowe and McKay 2016). The current study, however, used a different mindfulness measure (BMM; Berry et al. 2010), and questions were not open to such interpretation (e.g. ‘When I have distressing thoughts or images, I just notice them and let them go’).

The current findings highlight three mindfulness facets, encompassing the attentional component (acting with awareness) and attitudinal component (nonjudgment and nonreactivity) of mindfulness that are related to reduced frequency and distress in relation to OIT experience, and one facet (observe) that is related to increased frequency and distress. Individuals who are high in nonjudgment, nonreactivity and act with awareness facets of mindfulness experience fewer OITs. The attentional component of mindfulness (act with awareness) reflects an ability to focus on the task at hand, which would suggest that individuals who score high on acting with awareness may be less prone to intrusions, such as OITs. The attitudinal facets of mindfulness (nonjudgment and nonreactivity) reflect an opposing approach toward internal experiences to the negative judgments and reactions that are theorised to contribute to the persistence and distress associated with OITs in OCD (Rachman 1997, 1998, Salkovskis 1985). Indeed, in support of this idea, nonjudgment and nonreactivity also predicted less dysfunctional appraisals of OITs and less severe emotional reactions to OITs.

Mindfulness may offer an alternative method of responding to OITs to many of those carried out by individuals with OCD. In the present study, we found that nonjudgment, nonreactivity and act with awareness predicted less perceived difficulty controlling OITs and less use of control strategies. This seemingly contradictory finding highlights a key maintaining factor of control strategies in maintaining OIT experience (e.g. frequency and distress). Current cognitive theory of OCD suggests that the use of control strategies, such as thought suppression, contributes to distress related to OITs. When it comes to specific types of control strategy, we found that mindfulness related more strongly to cognitive thought control (e.g. thought suppression) than overt or behavioural (e.g. overt distraction, compulsions) control strategies. This is not surprising when considering that mindfulness is a state of mind that provides a cognitive method of responding to thoughts and feelings. Previous research indicates that use of thought suppression can lead to a rebound effect in OITs (e.g. Purdon 2004; Purdon et al. 2007), such that the individual would perceive a lack of control of their thoughts. Indeed, a meta-cognitive understanding of OCD suggests that appraisals regarding a lack of control over thinking are a key in maintaining symptomology (e.g. Wells and Papageorgiou 1998). The observed finding that the same mindfulness facets predict less difficulty in controlling OITs and less use of control strategies is consistent with our current understanding of distress relating to OITs. When viewed as a response strategy to OITs, an attitude of acceptance (nonreactivity and nonjudgment in particular) could offer an alternative approach to the more effortful and often ineffective control strategies assessed here. In this regard, increased mindfulness is related to decreased use of control strategies that contribute to the rebound of OITs (increased frequency, increased distress). This is important to note when considering that individuals with OCD report carrying out more control strategies than nonclinical individuals (García-Soriano and Belloch 2013). Future research should explicitly test whether decreased use of effortful control strategies, and increased mindfulness, can change the individual’s perceived control over OITs (less difficulty in controlling).

As predicted, the observe facet (e.g. ‘I intentionally stay aware of my feelings’) of mindfulness appears to be related to more negative experiences of OITs. Individuals who tend to observe their internal experiences reported a greater frequency of OITs, which may reflect a true increased frequency or an increased awareness of their occurrence. This tendency to observe experiences is also related to how individuals respond to their OITs, with a tendency toward negative reactions and appraisals and ineffective strategies. Although learning to ‘observe’ OITs may be a crucial step in learning an alternative approach to responding, it has been argued that some OITs are inherently distressing (Cougle and Lee 2014). Increasing an individual’s ability to observe inherently distressing thoughts could therefore be considered inappropriate, particularly if the other aspects of mindfulness (e.g. nonjudgment) are not adequately taught or practiced. Overall, these findings indicate important directions for future research investigating mindfulness-based interventions in the context of OCD. This study and previous research (Fairfax 2008) indicate that there may be specific facets of mindfulness that will be most useful in the context of OCD. Some researchers have suggested that increasing an individual’s ability to suspend judgment of internal experiences (nonjudgment) may be a particularly useful direction for mindfulness interventions for OCD (Watson and Purdon 2008). Indeed, nonjudgment is indicated as the most predictive mindfulness facet of psychopathology in general (Cash and Whittingham 2010). Preliminary research on the application of mindfulness-based interventions (e.g. mindfulness-based cognitive therapy, MBCT) with individuals with OCD also suggest that the attentional component and attitudinal component of mindfulness are important. In a qualitative analysis of MBCT for OCD, participants indicated that the most important aspects were learning to redirect attention away from OITs and to bring an attitude of acceptance toward those experiences (Hertenstein et al. 2012). Furthermore, small-scale intervention and experimental studies suggest that an attitude of acceptance (nonjudgment and ‘letting go’) mediates the effect of mindfulness interventions on reducing symptoms of OCD (Hanstede et al. 2008; Wahl et al. 2013). Crane et al. (2017) identify essential components for any mindfulness intervention, which highlight the attitudinal and attentional components. In particular, an ‘approach orientation’ is considered as a core component of mindfulness programmes, alongside present moment focus; self-regulation is enhanced by the cultivation of an ‘internal climate of friendliness toward experience’ (p. 5; Crane et al. 2017). Future intervention research should consider these guidelines and aim to investigate the effects of targeting specific mindfulness facets on the experience of OITs, which could determine potential benefits and harm. Building such evidence would then inform recommendations for clinical practice. Further experimental inductions of mindfulness and OITs in the laboratory could also extend our understanding to state experiences.

The current study is limited by the cross-sectional design, and sampling method. Causality cannot be assumed from our findings; the observed predictive relationships may reflect a bi-directional relationship between mindfulness and OITs. Replication in a prospective design could demonstrate the stability of the observed predictive relationships over time. In order to establish the direction of these relationships, experimental or intervention studies with samples of individuals who score high on OCD traits could assess whether tailored mindfulness meditations that emphasise attitudinal processes of acceptance (nonjudgment and nonreactivity) can mitigate the potential negative experience of OITs. The convenience sample recruited from a university setting limits the generalisability of the findings of the current study. A continuum approach to understanding the experience of OITs was adopted as a theoretical stance to this study, and therefore a convenience sample was deemed relevant. The distribution of scores of OCD symptomology, along with the high proportion of participants scoring over the clinical cut-off (32%), indicated that a broad range of experiences is represented in the current sample. The population sampled from a university setting is likely to be predominantly students, which may have skewed the findings. Differences in age were indicated between the clinical and subclinical groups that were created, and therefore age was entered as a covariate in subsequent preliminary analyses. Differences in OIT experience and mindfulness between the two groups were retained over and above the influence of age. Nonetheless, the findings of the current study require replication in clinical and community samples.

In summary, the present study investigated the predictive relationships between facets of trait mindfulness and experiences of obsessive intrusive thoughts. In support of our hypotheses, we demonstrated that specific facets of mindfulness relating to acting with awareness and acceptance (nonjudgment and nonreactivity) are related to less negative experiences of OITs. In contrast, increased levels of the observe facet of mindfulness may reflect a hypervigilance to OITs. Future research should investigate the effects of tailored mindfulness-based interventions for OCD to determine potential differential effects of increasing specific facets of mindfulness.
